# Clinical outcomes and cost-effectiveness of massage chair therapy versus basic physiotherapy in lower back pain patients: A randomized controlled trial

**DOI:** 10.1097/MD.0000000000019514

**Published:** 2020-03-20

**Authors:** Seung-Kook Kim, Aran Min, Chuljin Jeon, Taeyun Kim, Soohyun Cho, Su-Chan Lee, Choon-Key Lee

**Affiliations:** a Himchan UHS Spine and Joint Centre, University Hospital Sharjah, Sharjah, United Arab Emirates; b Joint and Arthritis Research, Orthopedic Surgery, Himchan Hospital, Seoul; c Department of Pharmaceutical Medicine and Regulatory Sciences, College of Medicine and Pharmacy, Yonsei University, Incheon, Republic of Korea; d Medical R&D Center, Bodyfriend, Seoul; e Department of Orthopedic Surgery, Joint Center, Busan Himchan Hospital, Busan, Republic of Korea.

**Keywords:** low back pain, massage chair, massage therapy, mechanical chair, physical therapy, physiotherapy

## Abstract

**Introduction:**

Low back pain is a chronic recurrent symptom, which can lower the patient's quality of life. With technological development of automated home massage systems, now offers a promising alternative to physiotherapy. However, thus far, the effectiveness of such methods has not been evaluated. We aimed to compare the efficacy and cost-effectiveness of a massage chair with those of conventional physiotherapy for the treatment.

**Methods:**

This was a randomized controlled trial with a two-group parallel design. Following randomization and allocation, 56 participants were enrolled to receive either physiotherapy (n = 25) or mechanical massage using the massage chair (n = 31). Pain severity was measured using a visual analog scale (VAS) and satisfaction assessed with the McGill Pain Questionnaire (MPQ). Quality of life modification was analyzed using the Functional Rating Index (FRI). Cost-effectiveness was analyzed by comparing the sum of physiotherapy fees and monthly rental fees for chair massage.

**Results:**

Physiotherapy and massage chair were both effective for pain control as assessed with the VAS (*P* < .001), satisfaction as assessed by MPQ (*P* < .001) and life quality improvement as assessed by FRI (*P* < .001) in both groups. Both VAS and FRI scores were significantly higher for physiotherapy than for massage chair (*P* = .03 and *P* = .03, respectively). There was no significant difference in MPQ between the two groups. Massage chair therapy was more cost-effective than physiotherapy, at only 60.17% of the physiotherapy cost (*P* < .001).

**Conclusions:**

The home massage chair system was cost-effective, but pain control and disability improved more with physiotherapy. However, our results showed that the massage chair is a promising treatment for pain control and quality of life modification, but efficacy is still superior in physiotherapy and the chair is not a replacement for physiotherapy.

**Trial registration:**

Clinical Research Information Service, KCT0003157. Retrospectively registered August 2, 2018.

## 1 Introduction

Lower back pain (LBP) is one of the common causes of disability and inability to work, and almost 70% to 75% of the population experience one attack of pain during their lives.^[1]^ This medical problem is compounded by the economic burden it imposes on patients.^[2,3]^ In the United States, the overall cost of treating LBP is more than 77 billion dollars; 13% of patients receive physiotherapy and the average cost is 11,151 dollars per person.^[4,5]^ 90% of all cases of LBP cases are of unknown etiology, with benign degenerative issues^[6]^ and only 5% to 10% of patients with discogenic nerve compression and spinal instability require surgical intervention.^[7]^ Treatment for LBP involves clinic-based physiotherapy which includes high thermal muscle relaxation, muscle stimulation, and ultrasound-based relaxation, all of which have demonstrated efficacy for pain control.^[7,8]^ Currently, symptomatic treatment, including various exercise and massage therapies, is a promising treatment strategy to relieve pain. However, there is insufficient evidence to support the efficacies of these alternative therapies.^[9]^ Relaxation massage and movement education, which can be beneficial to individuals who have back pain, have been thoroughly investigated. However, only the effectiveness and safety of vibration therapy have been assessed through observational studies.^[10,11]^ Thus far, the safety and effectiveness of multifunctional chair systems with heating, stretching, and relaxation functions have not been assessed. Furthermore, while such mechanical massage may reduce medical costs and increase accessibility of treatment, its cost-effectiveness has not yet been assessed. The purpose of the present study was to compare clinical outcomes such as pain control, satisfaction, and quality of life modification, as well as the cost-effectiveness of massage chair therapy with those of in-hospital physiotherapy.

## 2 Methods

### 2.1 Design overview and randomization

This was a prospective designed randomized controlled trial, and the evaluator was blinded. This study was approved by our institutional review board (Himchan-IRB 112294-01-201710-01) and is registered with the Clinical Research Information Service (KCT0003157; registered at August 2, 2018). We employed a two-group parallel design and calculated the required sample size for a comparative study using a two-sided *t* test using G-power for Windows (version 3.1.9.4; Brunsbuttel, Germany). Participants were recruited from an outpatient clinic and randomly assigned to one of two groups, either physiotherapy or mechanical massage chair therapy, using a confidential computer program (Phantom, Bodyfriend, Seoul, Korea). Written informed consent was obtained from all patients before starting therapeutic intervention.

### 2.2 Setting and participants

The study was conducted at Himchan Hospital, Busan, Korea. Participants were spine center outpatient clinic LBP patients between December 2017 and March 2018 and were enrolled and randomly assigned to treatment conditions. Inclusion criteria were as follows:

1) age 20 to 65 years,
2) body mass index (BMI) between 17 and 30kg/m^2^, and
3) no history of spine surgery.

A total of 61 patients with back pain were recruited after three weeks of advertising targeted to outpatients. Patients were excluded if they had pain radiating from the leg, cognitive impairment affecting the survey, recent vertebral fractures, serious comorbid underlying diseases, medication including non-steroidal anti-inflammatory drugs (NSAIDs), or evidence of progressive neurologic deficits. The evaluation was restricted to participants who completed the 3-week follow-up questionnaire and had been randomized to receive either massage chair therapy or physiotherapy. Finally, 31 (55.36%) participants were allocated to receive physiotherapy, and 25 (44.64%) were allocated to receive massage chair therapy.

### 2.3 Intervention

In each group, patients received up to 6 treatment sessions within 3 weeks. These were conducted by 9 accredited physiotherapists, each with a minimum experience of 2 years, and the intervention methods for LBP were authorized by the Korean Physiotherapy Association.

The massage chair therapy protocol was developed by three doctors employed by the manufacturer who developed the device (Bodyfriend, Inc., Seoul, Korea). Total massage time was 20 min, including 3 min of constant stretching, 5 min of vibration mode and stroke mode, and 40-degree heating of the treatment region. This protocol is called the “back strengthen mode” and is one of the machine's most commonly used programs.

The outpatient physiotherapy program was comprised of 5 min of ultrasound massage, 5 min of transcutaneous electrical nerve stimulation (TENS), 5 min of interferential current therapy, and 5 min of hot pack application. This approach is commonly used in patients for LBP.^[12]^

### 2.4 Data collection

Before randomization and group allocation, baseline characteristics such as age, height, body weight, BMI, vital signs, and history were recorded for all subjects. Subjective pain intensity was evaluated using the visual analog scale (VAS)^[13]^ and detailed characteristics of pain, including location, nature, and the patients’ satisfaction were assessed using the short-term McGill Pain Questionnaire (MPQ).^[14]^ Quality of life modification was assessed using the Functional Rating Index (FRI).^[15]^ Prior to the application of both therapies, as well as at 1, 2, and 3 weeks after the initiation of therapies all of the above-mentioned parameters were recorded. Following the collection of all data, patients were asked their opinions about the overall outcomes of their treatment. The cost of physiotherapy was calculated as the sum of the cost that was covered under the national health insurance system and the cost borne by the patient. The massage chair therapy cost was derived from the monthly rental fee charged by Bodyfriend.

### 2.5 Scales

The first measures are the VAS score and frequency of pain.^[13]^ Patients were instructed to indicate the severity of their pain on a scale bar between “0” (no pain) and “10” (the most extreme pain experienced ever). We used a scale bar that was specifically designed for back pain evaluation. The advantages of this method are that it is statistically sensitive and can be applied to either individuals or a small group.

The second measure is the result of the MPQ score.^[14]^ The MPQ is a self-reporting questionnaire. It comprises three main question types regarding pain: sensation, emotion, and subjective pain. It selects the most appropriate word for each of the 20 questions presented and evaluates the severity of the pain on a scale of 1 to 5. It provides quantitative information about the degree of treatment and distinguishes pain reduction with greater sensitivity than other methods.

The third measure is the FRI score.^[15]^ The FRI measures quality of life, back pain, and radiating pain intensity on a scale of 0 to 10. Quality of life includes 8 parameters, sleeping, washing, traveling, lifting, working, performing hobbies, walking, and standing. In addition, the pain intensity and frequency are evaluated. Of a total of five points, “The scoring method is calculated as (total score/40) ∗ 100 and converted from 0 to 100, and a higher score means that the pain is severe, and physical functional capacity is compromised.^[16]^

### 2.6 Statistical analysis

Data are indicated as mean and standard deviation (mean ± standard deviation, SD). Differences in baseline information between groups were compared using an independent *t* test. Pre- and post-treatment VAS scores, MPQ results, FRI results and cost were compared using a two-sided Student's *t* test and the outcomes of each method were assessed via a paired *t* test and or Wilcoxon signed-rank test, as appropriate. All statistical analyses were performed using SPSS for Windows (version 22.0; SPSS, Inc., Chicago, IL). Statistical significance was set at a *P*-value of <.05.

## 3 Results

### 3.1 Patient baseline characteristics

Baseline information for patients is presented in Table 1. Mean participant age was 48.40 ± 9.52 years in the physiotherapy group, and 38.84 ± 9.68 years in the massage therapy group (*P* = .42). There were no significant differences between the two treatment groups in terms of gender (*P* = .22). The duration of pain was 10.52 ± 12.93 months in the physiotherapy group and 9.90 ± 10.15 months in the massage treatment group; the difference was not significant (*P* = .76).

**Table 1:**
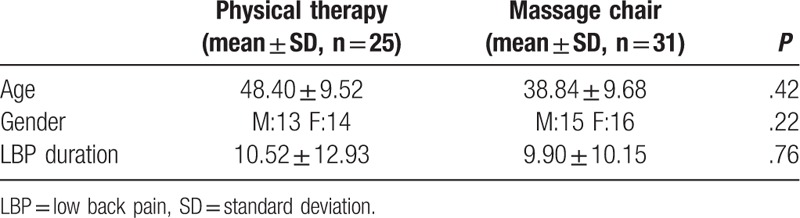
Patient baseline characteristics.

### 3.2 Effectiveness of treatment

Clinical outcomes, including the degree of pain reduction and quality of life modification for each group, are presented in Table 2. Comparison of pain before and after treatment using the VAS revealed that pain reduction was effective in both the physiotherapy and massage chair groups (*P* < .001 in both cases). When comparing pain differences in each group on the MPQ scale, there was significant pain reduction in both the physical therapy and massage chair groups (*P* < .001 in both cases). In terms of quality of life modification assessed using the FRI, both physiotherapy and massage chair were effective (*P* < .001 in both cases). No complications or aggravation of pain following treatment were reported.

**Table 2:**
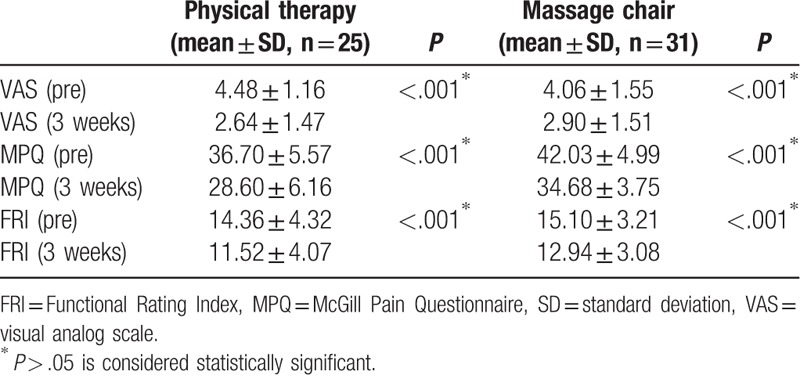
Effectiveness of pain relief evaluated by VAS, FRI, and MPQ.

### 3.3 Comparison of clinical outcomes between the intervention methods

**Figure 1:**
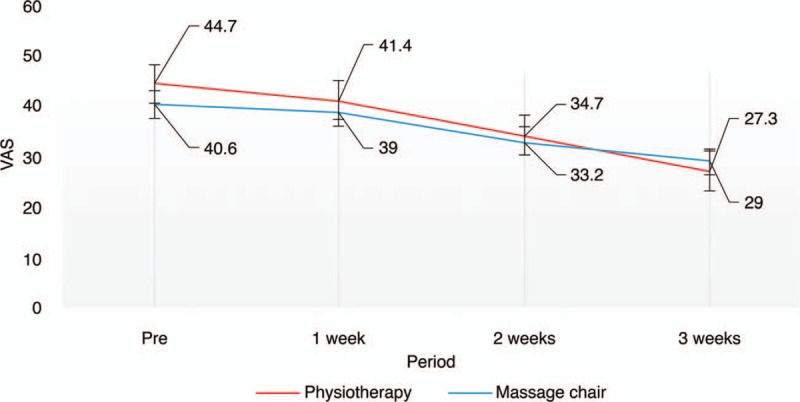
Visual analog scale (VAS) change during the 3-week treatment.

**Figure 2:**
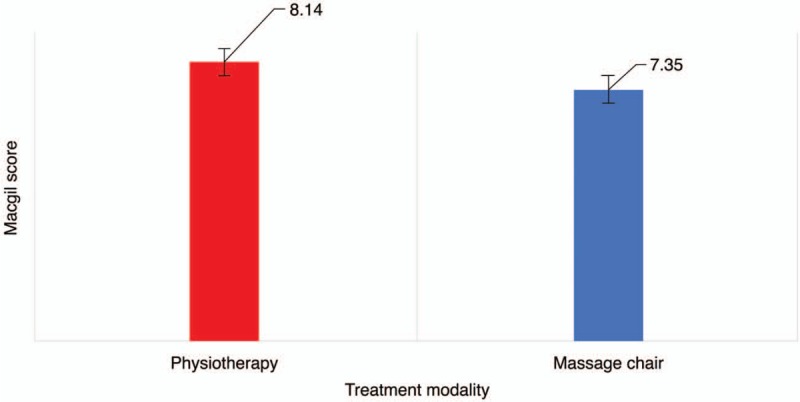
Changes in the McGill Pain Questionnaire (MPQ) score after 3 weeks of intervention.

**Figure 3:**
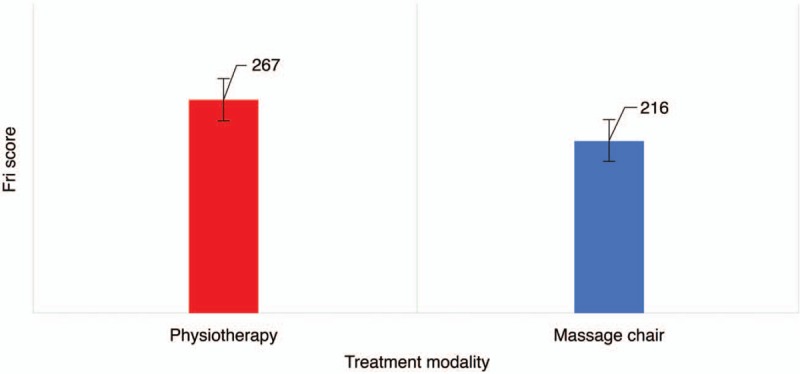
Functional Rating Index (FRI) improvement after 3 weeks of intervention.

The comparison of outcomes between the two groups is shown in Table 3. On the VAS scale, pain improvement in the physiotherapy group (1.73 ± 1.14) was significantly higher than that in the massage chair group (1.16 ± 0.78) (*P* = .03, Fig. 1). The MPQ score 3 weeks after treatment showed that massage chair therapy (8.14 ± 1.42) was more effective than physiotherapy (7.35 ± 2.24); however, this difference was not significant (*P* = .27, Fig. 2). A comparison of quality of life modification based on the FRI scale showed that physiotherapy (2.67 ± 1.85) was significantly more effective than massage chair therapy (2.16 ± 1.64; *P* = .03, Fig. 3).

**Table 3:**
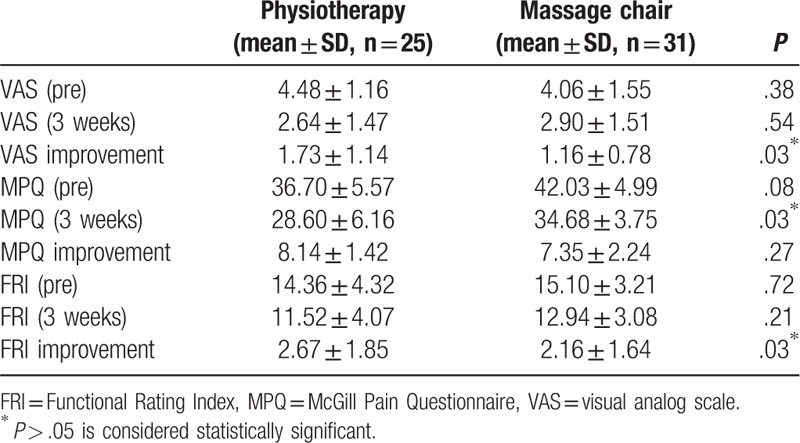
Differences in the degree of pain evaluated by VAS, FRI, and MPQ.

### 3.4 Cost of care

The total cost of physiotherapy was determined as the amount set by the national health insurance. The patients were charged 4.03 United States Dollars (USD) per physical treatment and 9.79 USD for health insurance; thus, the total cost of therapy was 166.82 USD per month (*P* < .001, Table 4). The monthly rental fee for the mechanical massage machine used in this study was 100.38 USD. Thus, the total cost of mechanical chair therapy was 60.17% of that of conventional physiotherapy.

**Table 4:**
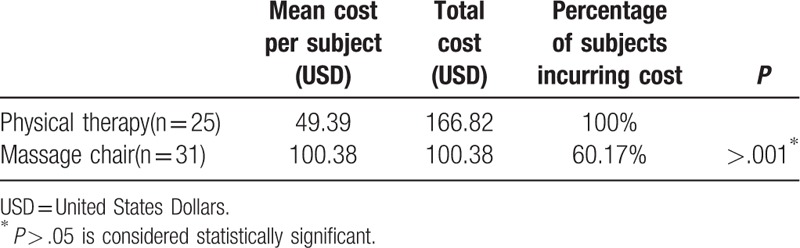
Mean costs of the treatments.

## 4 Discussion

The results of our study suggest that both clinic-based physiotherapy and mechanical massage chair demonstrate effectiveness in pain control, patient satisfaction, and life quality modification. Clinic-based physiotherapy demonstrated significant superiority in pain control and life quality modification. Alternatively, mechanical massage chair therapy was superior in terms of cost-effectiveness.

The Agency for Health Care Policy and Research guidelines by the United Kingdom Royal College of General Practitioners suggests that massage treatments are effective, but not affordable, for patients with back pain.^[17,18]^ However, the various technologies have been recently advanced and cost-effectiveness improved compared to the past. Additionally, we sought to validate the results of a previous study that demonstrated that massage chair treatment to be less effective than actual massage therapy.^[19]^ In our study, the massage chair was cost-effective and adequately controlled pain.

Massage therapy displays at least moderate continuous pain reduction compared to continuous stimuli in subacute, chronic pain patients and moderate enhancement in function.^[20,21]^ Furthermore, massage treatments are associated with a low rate of serious complications; only 13% of the population receiving massage therapy complained of therapy-related discomfort.^[21]^ Here, we used two treatment modes for pain control. The recovery and stretch modes involve rubbing and tapping designed to reduce dermal stimulation, which can help control pain based on gate control theory.

The results of our study suggested that both physiotherapy and mechanical chair therapy were effective in terms of pain reduction and overall quality of life modification. However, some measures of pain control and disability, such as the VAS and FRI, which reflect relatively diverse living improvements and pain control, showed greater pain reduction after clinic-based physiotherapy. Notably, massage chair therapy satisfaction as assessed with the MPQ was not inferior to that of physiotherapy, and the overall cost of mechanical massage therapy was lower than that of physiotherapy. These results indicate that mechanical massage chair therapy may be a clinically effective and cost-effective treatment method for LBP. Although this therapy may not yet be an authorized treatment or covered under many national insurance systems, our results support its effectiveness as an alternative to physiotherapy for back pain treatment. Both physiotherapy and mechanical massage chair therapy effectively reduced LBP.

The principles behind the two treatments are different. In clinic-based physiotherapy, extradermal heating therapy causes distension of the blood vessels in muscles around the spine, resulting in metabolic acceleration, increased flexibility of ligament tissue, and decreased pain.^[22]^ Laser therapy uses short-wavelength non-invasive light to restore anti-inflammatory activity and induce binding of tissues.^[23]^ TENS, which involves the use of an electrical current, has been suggested for pain reduction, but clinical evidence supporting its effectiveness is still lacking.^[24]^ Alternatively, the principle of mechanical massage therapy can be explained by the gate control theory.^[23]^ Back pain is transmitted through mechanical receptors on the skin to the spinal cord and back to the brain. During this process, when another sensory signal enters the spinal cord, the gates open or close before the signal is transferred to the brain. Massage creates a large number of sensory signals, which may either close or partially open the spinal cord nerve gates. Closed nerve gates prevent these stimulation signals from being transmitted to the central nervous system, thus blocking the path of the pain signal to the brain during massage. Therefore, it can be applied conveniently in everyday life and is cost-effective and accessible. Furthermore, our results indicate that massage chair therapy is effective in terms of quality of life modification.

There was a significant difference in the disability score between the two groups. Conventional physiotherapy was more effective than massage chair therapy as assessed by the FRI, which evaluates the emotional effects of pain and evaluates subjective overall pain intensity in a more detailed manner. Mechanical massage is a treatment method that can be administered by a machine without human contact. Finally, compared to physiotherapy, massage chair therapy requires no emotional support and human contact, and is associated with a lack of emotional connection between patients and the medical practitioner. This is one possible explanation for the reduced effectiveness in pain control and improvement for disability. However, massage chair machines can be useful in terms of cost-effectiveness and accessibility; therefore, while satisfaction with this treatment was inferior to physiotherapy, it was superior in terms of cost-effectiveness.

Mechanical massage resulted in pain control satisfaction and quality of life modification. Furthermore, because of technological developments, several systems to assess the health of the body, vibration function, and temperature control (including heating) may be developed for pain control. This may become a more patient-friendly method, as the machine might then converse and play music via artificial intelligence for people who do not prefer to sit alone in the mechanical massage chair. This would help to overcome the limitation of machines and would add to their existing benefits, such as easy accessibility and relatively low cost.

Our study is novel in that it is the first to make use of a prospective design and randomized control to compare the effectiveness of mechanical chair massage with conventional treatment. There were some limitations in our study. First, there is limited scope for generalization due to the small number of study participants and short follow-up duration. Although no complications were reported, our results should be interpreted with caution. Further studies with a multicenter trial design are needed to compare the efficacies of these two treatment modalities in a larger group of participants. Second, we did not identify the origin of pain in our study participants. However, our study design was prospective and strictly controlled. In addition, we used multiple clinical scales, which support the clinical significance of our findings. Also, this study has value in that it is the first trial of comparison between in-hospital management and mechanical chair treatment. In future studies, diagnosis using radiologic and clinical examinations should be performed before treatment. Despite these limitations, we are the first to attempt this trial and thereby demonstrate that mechanical massage therapy may have a therapeutic advantage in the treatment of LBP; large-scale, multicenter randomized controlled trials may corroborate the results of the present study.

## 5 Conclusions

The results of the present study suggest that physiotherapy remains superior for pain control and overall satisfaction relative to the massage chair; nonetheless, the massage chair is effective for pain control and patient satisfaction. Furthermore, satisfaction following treatment was not inferior while cost-effectiveness was superior after conventional physiotherapy. With technological development, mechanical systems may eventually provide promising treatment and large-scale studies have to be designed for continuous evaluation of this technology.

## Author contributions

**Conceptualization:** Seung-kook Kim.

**Data curation:** Seung-kook Kim, Aran Min.

**Formal analysis:** Aran Min, Chuljin Jeon.

**Methodology:** Taeyun Kim.

**Supervision:** Choon-Key Lee MD.

**Visualization:** Su-Chan Lee MD.

**Writing – review & editing:** Soohyun Cho.
